# Intraoperative Hypotension and Major Adverse Cardiac Events Among Older Adult Patients Undergoing Noncardiac Surgery: Retrospective Cohort Study

**DOI:** 10.2196/67177

**Published:** 2025-10-16

**Authors:** Kai Zhang, Chang Liu, Meng Wang, Ting Zhang, Bingbing Meng, Siyi Yao, Jingsheng Lou, Qiang Fu, Yanhong Liu, Jiangbei Cao, Lulong Bo, Weidong Mi, Hao Li

**Affiliations:** 1 Department of Anesthesiology, The First Medical Center, Chinese People’s Liberation Army General Hospital, 28th Fuxing Road, Haidian District, Beijing, 100853, China, 86 15010665099; 2 Medical School of Chinese People’s Liberation Army General Hospital, Beijing, China; 3 School of Medicine, Nankai University, Tianjin, China; 4 Department of Anesthesiology, Changhai Hospital, Naval Medical University, Shanghai, China; 5 National Clinical Research Center for Geriatric Diseases, Chinese People’s Liberation Army General Hospital, Beijing, China

**Keywords:** intraoperative hypotension, major adverse cardiac events, geriatric patients, threshold, mean arterial pressure

## Abstract

**Background:**

Intraoperative hypotension (IOH) is an important risk factor for major adverse cardiac events (MACE) in patients undergoing noncardiac surgery. However, the IOH threshold in older adult patients remains controversial.

**Objective:**

This study aimed to explore an appropriate IOH threshold in older adult patients to decrease the risk of MACE.

**Methods:**

This study involved older adult patients undergoing noncardiac surgery (age ≥65 y) from January 2012 to August 2019 in the Chinese People’s Liberation Army General Hospital (PLAGH; 35,262 patients) and Shanghai Changhai Hospital from January 2024 to December 2024 (13,418 patients). Univariate moving-average plots and multivariate restricted cubic splines were used to determine the IOH thresholds associated with an increased risk of MACE. The relationship between the IOH threshold and MACE was assessed using univariate and multivariate logistic regression analyses by 3 different hypotension exposure forms (duration, area, and time-weighted average mean arterial pressure [MAP]).

**Results:**

Out of 35,262 patients, 874 developed MACE in PLAGH, and 296 of 13,418 patients developed MACE in Changhai Hospital. In PLAGH, MAP below an absolute threshold of 70 mm Hg was associated with MACE. When the IOH absolute threshold was 70 mm Hg, the risk of MACE demonstrated a “dose-increasing” effect with changes in IOH exposure, and the risk of MACE was significantly increased when the duration lasted >15 minutes (odds ratio 1.51, 95% CI 1.22-1.88; *P*<.001). The stratified analysis showed that in patients younger than 80 years, when intraoperative MAP dropped below 70 mm Hg for more than 15 minutes, the odds ratio was 1.38 (95% CI 0.86‐2.28), *P*<.01. In Changhai hospital, intraoperative MAP <70 mm Hg was also significantly associated with MACE. Furthermore, IOH lasting longer than 15 minutes substantially increased the risk of MACE.

**Conclusions:**

For older adult patients undergoing noncardiac surgery, intraoperative MAP should be kept above 70 mm Hg to reduce the risk of postoperative MACE.

## Introduction

Each year, more than 300 million surgical procedures are conducted globally, with noncardiac surgeries accounting for 85% of the total [1]. With the global rise in the aging population, there has been a corresponding increase in the number of older adult surgical patients, leading to a higher incidence of postoperative complications [2]. Previous studies have suggested that major adverse cardiac events (MACE) occur in more than 2% of patients and are responsible for one-third of postoperative deaths [3-5]. Because of the decreased physiologic reserve and multiple coexisting diseases in older adult patients [5,6], the risk of postoperative MACE in these patients is higher and needs more attention.

Intraoperative hypotension (IOH) is reportedly associated with postoperative cardiovascular complications and mortality in patients undergoing noncardiac surgery, and the association becomes magnified as the severity of hypotension increases [7-10]. With the continuous advancement of medical technology, recent years have witnessed the emergence of numerous intervention measures aimed at preventing IOH. One notable example is the intraoperative hypotension prediction index developed by Hatib et al [11], which uses machine learning algorithms for its construction. Clinical studies have substantiated the efficacy of this index in reducing the incidence of IOH [12-14]. However, further research is warranted to ascertain whether its application can also lead to a reduction in postoperative complications. Concurrently, the majority of studies using the hypotension prediction index have established a hypotension threshold of 65 mm Hg of mean arterial pressure (MAP) [12,13]. This threshold is largely derived from research conducted on adult patients. However, older adult patients exhibit reduced cardiopulmonary reserve capacity, diminished autonomic nervous system self-regulation, and a cardiovascular system that is often in a precarious state of equilibrium. Therefore, the applicability of this threshold to older adult patients remains a subject of debate. Establishing an accurate threshold for hypotension is crucial in optimizing older adult patient outcomes.

Therefore, this study was performed to explore the IOH threshold and its relationship with MACE in older adult patients undergoing noncardiac surgery.

## Methods

### Study Population

Perioperative data for patients required in this study were obtained from the First Medical Center of Chinese People’s Liberation Army General Hospital (PLAGH) and Shanghai Changhai Hospital, respectively. Perioperative data from the First Medical Center (January 2012 to August 2019) involved older adult surgical patients and were primarily used for exploring IOH thresholds associated with increased risk of MACE in this population. Data from Shanghai Changhai Hospital (January 2024 to December 2024) were specifically used for validating IOH thresholds in older adult surgical patients.

### Ethical Considerations

The Research Ethics Committee of the Chinese PLAGH approved this study (approval reference S2019-311-02). The requirement for informed consent was waived because this retrospective cohort study posed minimal risk to participants. The patients’ names and ID numbers were anonymized during data collection.

### Inclusion and Exclusion Criteria

Patients aged ≥65 years, who were scheduled to undergo noncardiac surgery under anesthesia, were included in this study. The exclusion criteria were nongeneral anesthesia, American Society of Anesthesiologists (ASA) physical status classification of >IV, hysteroscopic or body surface surgery [15], surgery duration of ≤30 minutes, incomplete clinical data, no intraoperative blood pressure values at 30-second intervals, and missing data on patient characteristics.

### Primary Outcomes

The primary outcome was MACE, defined as the composite of acute myocardial infarction (MI), unstable angina, heart failure (HF), new-onset severe arrhythmia, nonfatal cardiac arrest, and cardiac death, during the operation and within 30 days after noncardiac surgery [16,17]. Patients with MACE were identified by examining their medical records. For data elements in the patients’ medical records, we used structured query language, a standard language for storing, manipulating, and retrieving data from databases, to identify instances of data domains. We extracted the patients’ postoperative biochemical test results, electrocardiograms (ECGs), coronary angiograms, postoperative course descriptions, and consultation records describing the specific MACE. A patient could have more than one MACE. The complications were independently evaluated by 3 experienced doctors. Any disputes were resolved through discussion and negotiation.

MI [18] was defined as an increase and gradual decrease in troponin levels accompanied by at least one of the following: ischemic symptoms, abnormal Q waves on ECG, ST-segment elevation or depression, or coronary artery intervention (ie, coronary angioplasty). HF [17] was defined as new in-hospital signs or symptoms of dyspnea or fatigue, orthopnea, paroxysmal nocturnal dyspnea, increased jugular venous pressure, pulmonary rales on physical examination, cardiomegaly, or pulmonary vascular engorgement. New-onset severe arrhythmia was defined as ECG evidence of atrial flutter, atrial fibrillation, ventricular tachycardia, ventricular fibrillation, and second- or third-degree atrioventricular conduction block. Nonfatal cardiac arrest was defined as the absence of a cardiac rhythm or the presence of a chaotic rhythm requiring any component of basic or advanced cardiac life support. Cardiac death was defined as any death unless an unequivocal noncardiovascular cause could be established.

### Data Collection

The following preoperative and intraoperative variables related to MACE were collected [19]. First, demographic variables (age, sex, and BMI) and smoking and drinking status. Smoking (drinking) status included nonsmoker (nondrinker), current smoker (drinker), and former smoker (drinker). Patients who had stopped smoking (drinking) for >1 year were considered former smokers (drinkers). Second, medical history (cerebrovascular disease, coronary heart disease, valvular heart disease, HF, arrhythmia, hypertension, diabetes mellitus, conditions requiring cardiac interventions, or peripheral arterial disease) and cardiac interventions, including coronary artery bypass grafting and percutaneous coronary intervention. Third, the most recent laboratory test results before surgery (hemoglobin, serum creatinine [SCr], and fibrinogen). Fourth, preoperative medicine (antiplatelet drugs, β-blockers, angiotensin-converting enzyme inhibitors [ACEI] and angiotensin receptor blockers [ARB], diuretic, and statin). Finally, surgery-related variables (ASA score, surgery type, surgery duration, emergency surgery, blood loss, urine volume, crystalloid infusion, and blood pressure data). The blood pressure data included noninvasive and invasive blood pressure, which were recorded at 30-second intervals from the Madison database. If no invasive blood pressure measurement was obtained during the operation, noninvasive blood pressure data were recorded. The baseline MAP was defined as the patients’ mean preoperative MAP. Artifactual data were removed using previously published criteria; for instance, an invalid MAP value was defined as (1) systolic blood pressure of ≥300 or ≤20 mm Hg, (2) systolic blood pressure ≤ diastolic blood pressure +5 mm Hg, (3) diastolic blood pressure of ≤5 or ≥225 mm Hg, or (4) MAP of >155 or <25 mm Hg [20].

### Statistical Analysis

The patients were categorized by whether they had developed MACE. Continuous variables are expressed as mean and SD if normally distributed, and as median and IQR if nonnormally distributed. Categorical data are presented as frequency (%). For comparison of differences between the 2 groups, the variance test or Kruskal–Wallis rank-sum test was used for continuous variables, and the chi-square test or Fisher exact test was used for categorical variables, as appropriate.

Moving-average smoothing plots with widths of 1, 3, and 5 minutes were used to determine the lowest MAP. Then, as previously reported [9], the linearity between each MAP exposure and outcome was modeled by a restricted cubic spline function with 3 knots located at the 10th, 50th, and 90th percentiles, and univariate moving-average plots and multivariate smoothed cubic spline curves were used to determine the IOH threshold associated with an increased risk of MACE. The confounding variables were determined from the variables with a standard mean difference of <0.2 in the baseline and with clinical meaning. These confounding variables included age, cerebrovascular disease, coronary heart disease, valvular heart disease, HF, arrhythmia, hypertension, peripheral arterial disease, renal insufficiency, hemoglobin, SCr, fibrinogen, antiplatelet drugs, β-blockers, ACEI, ARB, diuretic, ASA score, surgery type, surgery duration, emergency surgery, blood loss, urine volume, and crystalloid infusion.

Next, univariate and multivariate logistic regression analyses were performed to validate the practical value of IOH threshold by three different IOH exposure methods as described in the previous study: (1) time spent at the threshold associated with harmful outcomes, (2) total area under the threshold–time plot, and (3) time-weighted average mean arterial pressure (TWA-MAP) under the threshold. After that, stratification analysis was used to explore whether the association between the IOH threshold and the occurrence of postoperative MACE differed between the very old (≥80 y old) and older adults.

Except where noted, a 2-tailed *P* value of<.05 indicated a significant difference. All analyses were performed using R version 3.6.3 (The R Foundation for Statistical Computing).

## Results

### Patient Characteristics and Confounding Variables in PLAGH

**Figure 1.**
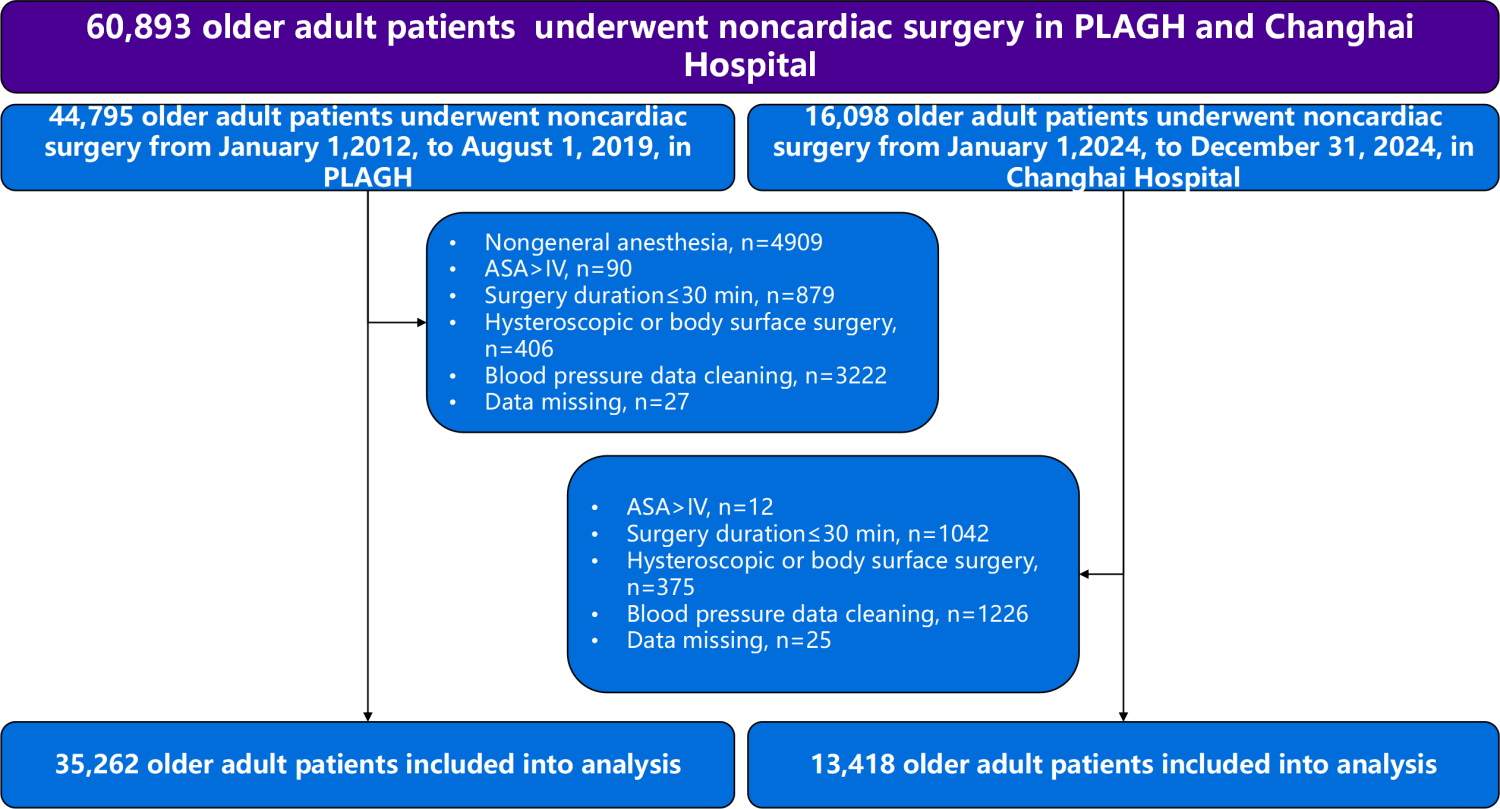
Patient flowchart. ASA: American Society of Anesthesiologists; PLAGH: People’s Liberation Army General Hospital.

In total, 44,795 older adult patients (≥65 years old) undergoing noncardiac surgery were identified from January 1, 2012, to August 1, 2019. Of these patients, 35,262 were included in the study after application of the inclusion and exclusion criteria (Figure 1), and 874 (2.48%) patients developed MACE. Of these 874 patients, 101 (0.29%) had acute MI, 139 (0.39%) had HF, 391 (1.11%) had arrhythmia, 215 (0.61%) had angina, and 65 (0.184%) had nonfatal cardiac arrest or cardiac death. There were significant differences between patients with and without MACE in terms of the ASA grade, hypertension, arrhythmia, HF, coronary artery disease, valvular heart disease, peripheral artery disease, hemoglobin concentration, SCr concentration, fibrinogen concentration, statin therapy, antiplatelet drugs, diuretics, type of surgery, surgery duration, colloid use, blood loss, and urine volume. However, there was no difference in the baseline MAP between the 2 groups (Table 1).

**Table 1.** Patients’ baseline and intraoperative characteristics in People’s Liberation Army General Hospital.

Demographic characteristics, variable, and level	Non-MACE^a^ (n=34,388)	MACE (n=874)	*P* value	SMD^b^
Sex, n (%)			.54	0.02
Female	16527 (48.1)	410 (46.9)		
Male	17861 (51.9)	464 (53.1)		
Age (y), mean (SD)	71 (5.11)	73 (6.11)	<.001	0.40
Age≥80 y, n (%)	2392 (7)	132 (15.1)	<.001	0.26
BMI^c^ (kg/m^2^), median (IQR^d^)	24.4 (22.2-26.8)	24.3 (22.1-26.8)	.28	0.04
Smoking status, n (%)			.15	0.06
Nonsmoker	32249 (93.8)	806 (92.2)		
Current smoker	1079 (3.1)	36 (4.1)		
Former smoker	1060 (3.1)	32 (3.7)		
Drinking status, n (%)			.16	0.06
Nondrinker	27732 (80.6)	696 (79.6)		
Current drinker	4553 (13.2)	111 (12.7)		
Former drinker	2103 (6.1)	67 (7.7)		
Previous history, n (%)				
Hypertension	15243 (44.3)	472 (54)	<.001	0.20
Diabetes	7845 (22.8)	231 (26.4)	.01	0.08
Arrhythmia	3226 (9.4)	207 (23.7)	<.001	0.39
Congestive heart failure	66 (0.2)	44 (5)	<.001	0.31
Coronary heart disease	3802 (11.1)	219 (25.1)	<.001	0.37
Valvular heart disease	148 (0.4)	24 (2.8)	<.001	0.19
Peripheral artery disease	750 (2.2)	48 (5.5)	<.001	0.17
Cerebrovascular disease	2649 (7.7)	103 (11.8)	<.001	0.14
Renal insufficiency	423 (1.2)	43 (4.9)	<.001	0.22
Preoperative laboratory data				
Hgb^e^ (g/L), median (IQR)	130 (119-141)	127 (111-139)	<.001	0.24
SCr^f^ (umol/L), median (IQR)	70.5 (60.0-82.5)	72.4 (61.2-88.0)	<.001	0.28
FB^g^ (g/L), median (IQR)	3.4 (2.9-4.0)	3.6 (3.0-4.5)	<.001	0.23
Preoperative medication, n (%)				
Statin	1862 (5.4)	89 (10.2)	<.001	0.18
Antiplatelet drugs	2886 (8.4)	108 (12.4)	<.001	0.13
β-blockers	2956 (8.6)	159 (18.2)	<.001	0.29
ACEI^h^	1306 (3.8)	51 (5.8)	.003	0.10
ARB^i^	3132 (9.1)	109 (12.5)	.001	0.10
Diuretic	2479 (7.2)	105 (12)	<.001	0.16
Surgery-related factors				
ASA^j^, n (%)			<.001	0.49
I	579 (1.7)	7 (0.8)		
II	26963 (78.4)	522 (59.7)		
III	6598 (19.2)	288 (33)		
IV	248 (0.7)	57 (6.5)		
Emergency surgery, n (%)	840 (2.4)	63 (7.2)	<.001	0.22
Type of surgery, n (%)			<.001	0.46
Ear and nose	1681 (4.9)	19 (2.2)		
Hepatobiliary	5885 (17.1)	139 (15.9)		
Orthopedics	9567 (27.8)	192 (22)		
Urology	3273 (9.5)	52 (6)		
General	6008 (17.5)	210 (24)		
Neurosurgery	1569 (4.6)	42 (4.8)		
Thoracic	2594 (7.5)	133 (15.2)		
Vascular	928 (2.7)	58 (6.6)		
Others	2883 (8.4)	29 (3.3)		
Surgery duration (min)			<.001	0.46
0-60	889 (2.6)	37 (4.2)		
60-180	17102 (49.7)	272 (31.1)		
180-240	7725 (22.5)	213 (24.4)		
240-infinity	8672 (25.2)	352 (40.3)		
Baseline MAP^k^ (mm Hg), median (IQR)	96 (89-103)	95 (87-102)	.04	0.07
Crystalloid infusion rate (ml/kg·min), mean (SD)	0.05 (0.0)	0.051 (0.0)	<.001	0.16
Blood loss (mL), median (IQR)	100 (50-200)	200 (100-300)	<.001	0.21
Urine volume (mL), median (IQR)	300 (100-550)	400 (200-700)	<.001	0.25

a MACE: major adverse cardiac events.

b SMD: standard mean difference.

c BMI; body mass index

d IQR; interquartile range

e Hgb: hemoglobin.

f SCr: serum creatinine.

g FB: fibrinogen.

h ACEI: angiotensin-converting enzyme inhibitors.

i ARB: angiotensin receptor blockers.

j ASA: American Society of Anesthesiologists.

k MAP: mean arterial pressure.

### Determination of IOH Thresholds

**Figure 2.**
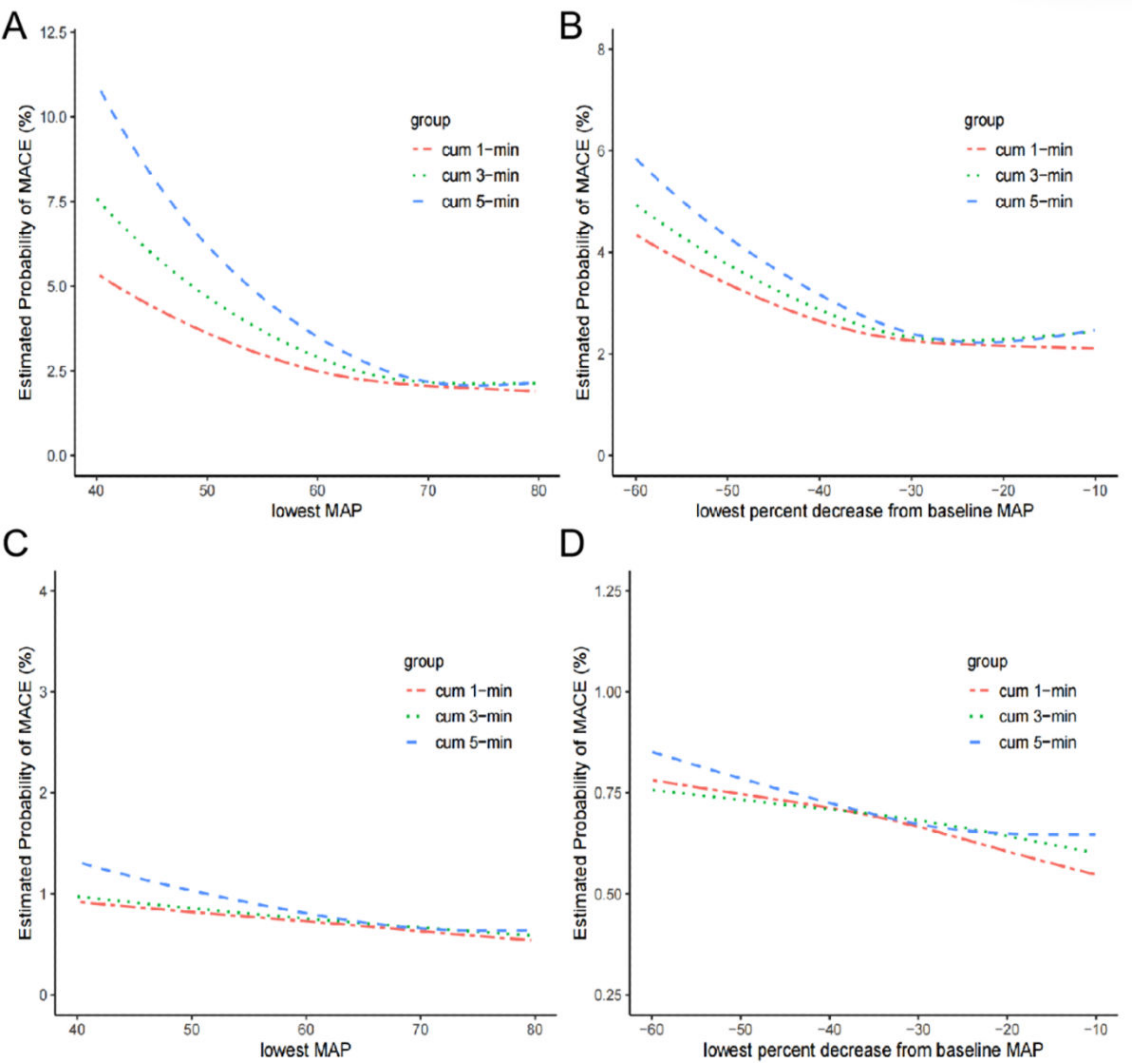
Univariable and multivariable relationship between MACE and absolute and relative lowest MAP thresholds. (**A, B**) Estimated probability of MACE was determined from the univariable moving-average smoothing plots. (**C, D**) Estimated probability of MACE was determined from smoothed multivariable logistic regression. MACE: major adverse cardiac events; MAP: mean arterial pressure; cum: cumulative.

As illustrated in Figure 2A and B, the analysis of univariate moving average smoothing curves indicates that the curves converge at a MAP of 70 mm Hg and a 30% reduction from baseline values across various cumulative time intervals. This finding suggests that an absolute MAP below 70 mm Hg or a relative decrease of 30% from baseline may represent a critical threshold for IOH in older adult patients. After adjusting for confounding factors—including age, cerebrovascular disease, coronary heart disease, valvular heart disease, HF, arrhythmia, hypertension, peripheral arterial disease, renal insufficiency, hemoglobin, SCr, fibrinogen, antiplatelet drugs, β-blockers, ACEI, ARB, diuretic, ASA score, surgery type, surgery duration, emergency surgery, blood loss, urine volume, crystalloid infusion—Figures 2C and 2D reveal that the curves maintain convergence at the 70 mm Hg mark but lose convergence at the 30% reduction point, thereby underscoring the robustness of using an absolute MAP below 70 mm Hg as a threshold for IOH in older adult patients. To further validate this threshold’s efficacy, the study used 3 widely adopted indicators of IOH exposure—time below the threshold, area under the curve (AUC) beneath the threshold, and TWA-MAP—subjecting them to multivariate logistic regression analysis. The adjusted model demonstrates a significant positive correlation between the duration of MAP below 70 mm Hg and the incidence of MACE. Specifically, when the duration of IOH exceeds 15 minutes, the risk of MACE substantially increases after adjusting for all confounding variables (adjusted odds ratio [OR] 1.51, 95% CI 1.22‐1.88, *P*<.001). Notably, the AUC for the duration below the hypotensive threshold, measured in minutes×mm Hg, and the TWA-MAP also significantly affect MACE occurrence. As exposure intensity rises, the risk of MACE exhibits a graded increase, which further illustrates the dose-dependent effect of hypotension (Table 2).

**Table 2.** Univariable and multivariable relationship between mean arterial pressure thresholds and outcomes.

Duration of intraoperative hypotension	OR^a^ (95% CI)	Adjusted OR (95% CI)	*P* value
Time with MAP^b^<70 mm Hg (per min)	1.01 (1.00-1.01)	1.00 (1.00-1.01)	<.001
Time with MAP<70 mm Hg (min)			
0.5‐5	1.23 (0.94-1.60)	1.26 (0.96-1.61)	.10
5‐10	1.27 (0.97-1.65)	1.23 (0.94-1.62)	.13
10‐15	1.10 (0.81-1.49)	1.06 (0.78-1.45)	.72
˃15	1.69 (1.38-2.07)	1.51 (1.22-1.88)	<.001
Area under curve<70 mm Hg (min×mm Hg)			
Q1	1.19 (0.93-1.52)	1.20 (0.93-1.54)	.16
Q2	1.28 (1.01-1.63)	1.22 (0.95-1.57)	.12
Q3	1.46 (1.15-1.85)	1.37 (1.07-1.75)	.01
Q4	2.09 (1.67-2.61)	1.82 (1.43-2.32)	<.001
TWA^c^-MAP<70 mm Hg (mm Hg)			
Q1	1.35 (1.06-1.71)	1.30 (1.01-1.66)	.04
Q2	1.36 (1.07-1.73)	1.29 (1.01-1.66)	.04
Q3	1.48 (1.17-1.87)	1.42 (1.11-1.82)	.005
Q4	1.83 (1.46-2.29)	1.56 (1.22-1.99)	<.001

a OR: odds ratio.

b MAP: mean arterial pressure.

c TWA: time-weighted average.

As illustrated in Table 3, among patients aged <80 years, compared with intraoperative MAP less than 70 mm Hg durations ≤5 minutes, the ORs of intraoperative MAP less than 70 mm Hg durations of 5‐10 minutes, 10‐15 minutes, and >15 minutes were 1.11 (95% CI 0.85‐1.44), 0.86 (95% CI 0.62‐1.17), and 1.31 (95% CI 1.09‐1.59), respectively. Among patients aged ≥80 years, the ORs of intraoperative MAP less than 70 mm Hg durations of 5‐10 minutes, 10‐15 minutes, and >15 minutes were 1.27 (95% CI 0.66‐2.54), 1.28 (95% CI 0.58‐2.64), and 1.38 (95% CI 0.86‐2.28), respectively.

**Table 3.** Results of stratification analysis.

Stratifications	Time with MAP^a^<70 mm Hg (min)	OR^b^ (95% CI)	*P* value
Age <80	0.5‐5	Reference	Reference
Age <80	5‐10	1.11 (0.85‐1.44)	.43
Age <80	10‐15	0.86 (0.62‐1.17)	.34
Age <80	˃15	1.31 (1.09‐1.59)	<.01
Age ≥80	0.5‐5	Reference	Reference
Age ≥80	5‐10	1.27 (0.66‐2.54)	.42
Age ≥80	10‐15	1.28 (0.58‐2.64)	.52
Age ≥80	˃15	1.38 (0.86‐2.28)	.19

a MAP: mean arterial pressure.

b OR: odds ratio.

### Validation of the IOH Threshold for Older Adult Patients in Shanghai Changhai Hospital

A total of 13,418 older adult surgical patients were included at Changhai Hospital. Among them, 296 patients (2.2%) experienced MACE (Figure 1). The baseline characteristics of the patients are presented in Table S1 in Multimedia Appendix 1. Univariate logistic regression analysis revealed a significant association between IOH and MACE (OR 1.01, 95% CI 1.00‐1.01, *P*<.001). Following adjustment for confounding factors, including age, sex, history of cerebrovascular disease, history of coronary heart disease, history of valvular heart disease, history of HF, history of arrhythmia, history of hypertension, history of peripheral arterial disease, history of renal insufficiency, SCr, preoperative hemoglobin, preoperative fibrinogen, ASA grades, surgery duration, emergency surgery status, intraoperative blood loss, and intraoperative crystalloid infusion volume, the association between IOH and MACE remained robust (adjusted OR 1.01, 95% CI 1.00‐1.01, *P*<.001). Furthermore, we found that IOH lasting ≥15 minutes was associated with a significantly increased risk of MACE (adjusted OR 2.01, 95% CI 1.55‐2.59, *P*<.001; Table S2 in Multimedia Appendix 1). These results are consistent with findings from the PLAGH study.

## Discussion

### Principal Findings

In this study, we investigated and validated the correlation between IOH and MACE in older adult patients undergoing noncardiac surgery from PLAGH and Shanghai Changhai Hospital. The findings indicate that the risk of MACE in older adult patients increases as MAP decreases during noncardiac surgery. Specifically, when the lowest intraoperative MAP falls below 70 mm Hg, there is a significant increase in the risk of MACE. Using an intraoperative MAP of 70 mm Hg as the reference threshold for IOH, the risk of MACE demonstrates a dose-dependent increase. In addition, if the duration of intraoperative MAP below 70 mm Hg exceeds 15 minutes, the risk of MACE increases above 50%. Consequently, using a MAP of 70 mm Hg as the reference threshold for IOH could offer more valuable guidance for older adult patients.

The relationship between IOH and postoperative complications has been extensively investigated by researchers [9,21-26]. Previous studies have consistently demonstrated an association between the severity of IOH and postoperative complications [8,27-30]. This severity encompasses not only the extent of the reduction but also the duration of hypotension and the area under the hypotension threshold. In line with previous studies, the results of our study demonstrate a strong correlation between the severity of IOH, as measured by time, AUC, or TWA-MAP, using a reference threshold of MAP 70 mm Hg, and the occurrence of MACE. Notably, our study also found that when MAP falls below 70 mm Hg for more than 15 minutes during surgery, the risk of MACE increases significantly. This finding may have important clinical implications. In liver resection surgery, where hepatic inflow occlusion is often necessary, the determination of an appropriate occlusion time is crucial. Our observation that MAP below 70 mm Hg for more than 15 minutes is associated with a 50% increased risk of MACE can serve as a valuable reference point in this context.

However, our study differs from previous research in terms of the currently accepted threshold for IOH. While the most used IOH threshold is a MAP of 65 mm Hg, our study suggests that a higher threshold of 70 mm Hg may be more suitable for older adult surgical patients. This discrepancy may be attributed to the fact that previous studies encompassed a general population, including patients aged 18 years and older or 45 years and older, whereas our study specifically focused on individuals aged 65 years and older. As mentioned earlier, the cardiovascular system of older adult patients is often delicately balanced, and even minor fluctuations in hemodynamics can have irreversible and severe consequences.

It is important to note the physiological differences in the very older adult population (aged 80 years and older). Within this cohort, blood pressure variability tends to be greater, necessitating more cautious monitoring and management during surgery. Although our study accounted for various confounding factors that may affect blood pressure fluctuations and the occurrence of postoperative MACE, the limitations inherent to retrospective studies prevented the adjustment for all potential confounders. As a result, our analyses did not yield sufficient statistical significance in this very old group. The specific factors contributing to the lack of significant findings could include the unique cardiovascular responses and decreased physiologic reserves that characterize this demographic. Our results underscore the need for future studies to refine the thresholds for IOH in older patients, as traditional metrics may not adequately reflect the risks faced by this vulnerable population.

This study has 2 main limitations. First, the occurrence of MACE was lower than that in other research, and this was primarily attributed to the limitations inherent in retrospective studies. The reliance solely on patients’ medical records for the identification of postoperative MACE may have led to underrecognition of specific cases of acute MI because of the absence of postoperative troponin monitoring. Second, although this study showed that a 25% decrease from baseline MAP was associated with MACE, no significant association was found in the subsequent validation analyses. This was also probably due to the limitations inherent in retrospective studies.

### Conclusion

For older adult patients undergoing noncardiac surgery, intraoperative MAP should be kept above 70 mm Hg to reduce the risk of postoperative MACE. However, for patients older than 80 years, further research is needed to explore the accurate threshold for IOH.

## Supplementary material

10.2196/67177 Multimedia Appendix 1 Supplementary material about Changhai Hospital.

## Abbreviations

ACEI: angiotensin-converting enzyme inhibitor
ARB: angiotensin receptor blocker
ASA: American Society of Anesthesiologists
AUC: area under the curve
ECG: electrocardiogram
HF: heart failure
IOH: intraoperative hypotension
MACE: major adverse cardiac event
MAP: mean arterial pressure
MI: myocardial infarction
OR: odds ratio
PLAGH: People’s Liberation Army General Hospital
SCr: serum creatinine
TWA-MAP: time-weighted average mean arterial pressure
