# Supplementary material for: Intraoperative Hypotension and Major Adverse Cardiac Events Among Older Adult Patients Undergoing Noncardiac Surgery: Retrospective Cohort Study
Source: JMIR Aging. 2025 Oct 16;8:e67177. doi: 10.2196/67177 (PMC12530642; doi:10.2196/67177)
Supplement: Multimedia Appendix 1 [file aging-v8-e67177-s001.docx]

**Table S1.** Patients’ baseline and intraoperative characteristics in Changhai hospital.

| Variable | | level | Non-MACE  (n=13122) | MACE  (n=296) | *P* |
| --- | --- | --- | --- | --- | --- |
| **Demographic characteristics** | | |  |  |  |
| Sex, n (%) | | female | 4543(34.6) | 101(34.1) | 0.907 |
|  | | male | 8579(65.4) | 195(65.9) |  |
| Age (yr), median [IQR] | |  | 71(68,75) | 72(68,75) | 0.028 |
| **Previous history****, n (%)** | | |  |  |  |
| Hypertension | |  | 6488(49.4) | 154(52) | 0.412 |
| Arrhythmia | |  | 612(4.7) | 21(7.1) | 0.070 |
| Congestive heart failure | |  | 45(0.3) | 2(0.7) | 0.278 |
| Coronary heart disease | |  | 1225(9.3) | 28(9.5) | 0.999 |
| Valvular heart disease | |  | 181(1.4) | 10(3.4) | 0.009 |
| Peripheral artery disease | |  | 1133(8.6) | 27(9.1) | 0.849 |
| Cerebrovascular disease | |  | 1528(11.6) | 39(13.2) | 0.472 |
| Renal insufficiency | |  | 173(1.3) | 5(1.7) | 0.768 |
| **Preoperative laboratory data** | | |  |  |  |
| Hgb (g/L), median [IQR] | |  | 132(121,138) | 128(120,136) | <0.001 |
| SCr (umol/L), median [IQR] | |  | 72(64,83) | 80(65,82) | 0.008 |
| FB (g/L), median [IQR] | |  | 3.01(2.74,3.6) | 3.36(2.8,3.6) | <0.001 |
| **Surgery-related factors** | | |  |  |  |
| ASA, n (%) | Ⅰ | | 91(0.7) | 0 | 0.025 |
|  | Ⅱ | | 8719(66.4) | 178(60.1) |  |
|  | Ⅲ | | 4077(31.1) | 109(36.8) |  |
|  | Ⅳ | | 235(1.8) | 9(3) |  |
| Emergency surgery, n (%) | |  | 1121(8.5) | 23(7.8) | 0.715 |
| Surgery duration (min), median [IQR] | | [0,60) | 2.17(1.33,3.17) | 2.33(1.58,3.42) | 0.006 |
| Crystalloid infusion volume (ml), median [IQR] | |  | 1600(1110,2270) | 1655(1140,2500) | 0.088 |
| Duration of intraoperative hypotension (min), median [IQR] | |  | 0(0,5) | 0(0,30) | <0.001 |
| Duration of intraoperative hypotension≥15 min, n (%) | |  | 2773(21.1) | 105(35.5) | <0.001 |
| Blood loss (ml), median [IQR] | |  | 50(20,100) | 50(30,200) | 0.007 |

MACE, major adverse cardiac events; Hgb, hemoglobin; SCr, serum creatinine; FB, fibrinogen; ASA: American Society of Anesthesiologists; IQR, interquartile range.

**Table S2**. The univariate and multivariate logistic regression analysis exploring the association of intraoperative hypotension with major adverse cardiac events (MACE) in Changhai hospital.

|  | Univariate logistic regression | |  | Multivariate logistic regression | |
| --- | --- | --- | --- | --- | --- |
|  | OR (95% CI) | *P* |  | OR (95% CI) | *P* |
| Time with MAP<70 mmHg (per min) | 1.006 (1.004-1.008) | <0.001 |  | 1.005 (1.003-1.007) | <0.001 |
| Time with MAP<70 mmHg |  |  |  |  |  |
| <15 min | 1 (ref) |  |  | 1 (ref) |  |
| ≥15 min | 2.052 (1.606-2.607) | <0.001 |  | 2.006 (1.548-2.589) | <0.001 |
